# Egocentric distance perception in older adults: Results from a functional magnetic resonance imaging and driving simulator study

**DOI:** 10.3389/fnagi.2022.936661

**Published:** 2022-10-06

**Authors:** Luis Eudave, Martín Martínez, Elkin O. Luis, María A. Pastor

**Affiliations:** ^1^Neuroimaging Laboratory, Division of Neurosciences, Centre for Applied Medical Research, University of Navarra, Pamplona, Spain; ^2^School of Education and Psychology, University of Navarra, Pamplona, Spain

**Keywords:** egocentric distance perception, fMRI, driving, simulator, older adults

## Abstract

The ability to appropriately perceive distances in activities of daily living, such as driving, is necessary when performing complex maneuvers. With aging, certain driving behaviors and cognitive functions change; however, it remains unknown if egocentric distance perception (EDP) performance is altered and whether its neural activity also changes as we grow older. To that end, 19 young and 17 older healthy adults drove in a driving simulator and performed an functional magnetic resonance imaging (fMRI) experiment where we presented adults with an EDP task. We discovered that (a) EDP task performance was similar between groups, with higher response times in older adults; (b) older adults showed higher prefrontal and parietal activation; and (c) higher functional connectivity within frontal and parietal-occipital-cerebellar networks; and (d) an association between EDP performance and hard braking behaviors in the driving simulator was found. In conclusion, EDP functioning remains largely intact with aging, possibly due to an extended and effective rearrangement in functional brain resources, and may play a role in braking behaviors while driving.

## Introduction

Egocentric distance perception (EDP) refers to the distance perceived between the observer and the stimulus. The EDP of a stimulus or an object in a 3D scene is determined by a set of optical variables called distance cues (Foley, 1977). These include, but are not limited to, depth, size, perspective, contrast, texture, shadows, surface inclination, or even gravity (Clément et al., 2016). It is usually considered that within an image, objects are close and scenes are distant. Thus, distant objects or scenes would occupy the upper half of the visual field, while closer objects take the lower half in addition to the fovea.

There are two types of EDP tasks: those based on a verbal answer and those based on action. The first type requires a verbally emitted response and includes the calculation of distances in metric units. The second type implies an action directed to the visual information that was presented just before the action. A common example of the latter is walking blindfolded toward the position of a previously presented object. These tasks assume that the action is controlled by a visual representation of the physical space. Although the neurological substrate underlying both types of EDP tasks has not been studied, we can assume that both processes transform visual signals in different ways and therefore use different resources (Milner and Goodale, 2008).

These studies on EDP are usually carried out in “real” physical places, both outdoors (garden and street) and indoors (rooms and corridors). There are also digital modalities in which scenes and objects are shown on a computer screen: some use optical illusions to simulate depth within the scene (Amit et al., 2012), while others show pairs of images of the same stimulus but at different distances (Parkinson et al., 2014; Persichetti and Dilks, 2016). This modality, displayed on a computer screen, could prove useful when studying situations where the perception of great distances is necessary, such as when driving a car on a highway. Studies comparing performance between real-world and PC-generated tasks (including virtual reality) show mixed results: some have found differences in performance (Adamo et al., 2012; Kimura et al., 2017), while in others both modalities show similar patterns of underestimation (Creem-Regehr et al., 2005; Kunz et al., 2009; Li et al., 2011; Hettinger and Haas, n.d.). This is relevant, since an EDP task “virtual analog” is needed when exploring its neural basis using functional magnetic resonance imaging (fMRI).

Driving is a complex daily-life activity that makes use of several cognitive abilities. For instance, when evaluating on-road fitness-to-drive, the best discriminative tests employed were the Ergovision Movement Perception subtest and Useful Field of View (UFOW), which have good discriminative ability to predict the performance of older drivers on a driving simulator, the Benton Line Orientation Task, Clock Drawing task, Driver Scanning, and the UFOW divided and selective attention subtests. These tests evaluate different aspects of perception, cognitive flexibility, attention, and spatial construction (Mathias and Lucas, 2009). Other functions, such as other executive functions, memory, or perceptual abilities, are also required for driving (Whelihan et al., 2005; Mäntylä et al., 2009; León-Domínguez et al., 2017). Some of these cognitive abilities deteriorate with aging, but only deficits in visuospatial abilities have been consistently associated with impaired driving, both real and simulated, in older adults (Reger et al., 2004; Hoffman et al., 2005; Mathias and Lucas, 2009). Investigating the mediating mechanisms of visuospatial abilities in driving is important since its deficit has been associated with real-world crashes and performance in a driving simulator in older adults (Anderson et al., 2005) and patients with cognitive impairment (Apolinario et al., 2009).

The study of EDP in aging has been little explored. It has been found that while young volunteers underestimated distances, older volunteers over 70 years of age did not and, surprisingly, had better accuracy (Bian and Andersen, 2013). To explain these results, the authors argue that this effect may be due to the fact that older adults have more knowledge about the egocentric distance from real scenes (Zhou et al., 2016), as a result of greater experience throughout life. Another explanation is the participants’ “height” effect, in which older adults have reached their height for a longer time than younger adults and therefore can make better distance estimates, even after controlling for height (Ooi and He, 2007; Zhou et al., 2016).

Other studies have reported alternate results. Gajewski et al. (2015) obtained egocentric distance detection thresholds for each subject; the results showed thresholds to be higher in older volunteers than in younger adults. However, the distance perception accuracy between both groups was similar, even when allowed to take a longer glance and when multiple stimuli appeared instead of one. They attributed these higher thresholds in older adults as necessary to extract useful information from the scene. In general, they conclude that this function is not impaired in older adults, and that the representation of space formed from memory plays an important role in this group. A subsequent study found support for this hypothesis (Wallin et al., 2017). Finally, Ruggiero et al. (2016) found slower and less accurate responses to egocentric distance judgments in participants above 70 years old, although this might be dependent on variables, such as scene context and sex, may affect the perception of distances in older adults (Norman et al., 2018).

Overall, previous studies indicate that EDP might be preserved in older age; however, the brain mechanisms involved in this task remain unclear. To date, there are few functional neuroimaging studies that have studied the neural correlates of EDP, and none included an older adult population. Activation of the lateral occipital cortex was found when stimuli were presented close and the parahippocampal area of places when presented further away (Amit et al., 2012). Using the perception of egocentric distance specific to scenes, not only to images of objects, activity was found in the retrosplenial complex in addition to the occipital lateral cortex but not in the parahippocampal area of places (Persichetti and Dilks, 2016). The right inferior parietal lobe (IPL) and areas from the default mode network (DMN) have also been found to be activated in tasks where spatial, temporal, and social perception of distance is examined (for example, “a close friend” and “a year from now”), which reinforces its role as an integrator of the perception of distance in a broader sense (Parkinson et al., 2014; Peer et al., 2015). It is important to note that functional connectivity networks of EDP have not been explored so far.

Studying how these activations change as we age could be of practical relevance, since an increased activation might reflect an increased cognitive demand, which might degrade EDP performance when executed in a complex everyday situation, such as car driving, and not as an isolated laboratory paradigm (Venkatesan et al., 2018). This is important since distance perception is attributed to be a perceptual mechanism of driving behaviors that may prevent accidents, especially in driving conditions, such as nighttime or foggy weather (Cavallo et al., 2001, 2007).

To date, there are a few functional neuroimaging studies that have studied the neural correlates of EDP, and none included an older adult population. In general, results report activations from the occipital V3d/V3A areas, and further objects were in pairs of images with stimuli at different distances (Berryhill, 2009). Activation from the lateral occipital cortex was found when stimuli were presented close and from the parahippocampal area of places when presented further away (Amit et al., 2012). Using the perception of egocentric distance specific to scenes, not only to images of objects, activity was found in the retrosplenial complex in addition to the occipital lateral cortex but not in the parahippocampal area of places (Persichetti and Dilks, 2016). The right inferior parietal lobe (IPL) and areas from the default mode network (DMN) have also been found to be activated in tasks where spatial, temporal, and social perception of distance is examined (for example, “a close friend” and “a year from now”), which reinforces its role as an integrator of the perception of distance in a broader sense (Parkinson et al., 2014; Peer et al., 2015). It is important to note that functional connectivity networks of EDP have not been explored so far.

Therefore, in this study, we aimed to explore the neural correlates of EDP in older adults as compared to younger adults using fMRI, and how performance impacts both populations on a common activity of daily living like car driving using a driving simulator. Our hypotheses for this experiment are as follows: (1) EDP performance is comparable between young and older adults, (2) a difference will be observed in brain activity and connectivity between groups, and (3) there will be a connection between EDP performance and driving behavior.

## Materials and methods

### Participants

A total of 36 volunteers were recruited and classified into two groups: 19 young subjects (YS, 11 men, 30.5 ± 4.5 years old [descriptive results will continue to be presented in the mean ± standard deviation format] and a mean 8.8 years of driving experience) and 17 older subjects (OS, 14 men, 66.5 ± 4.7 years old and a mean of 44.7 years of driving experience). The sample size and our EDP task design were calculated using POBE (v1.1), a Matlab-based program for the optimal design of blocked experiments (Maus and van Breukelen, 2014).

Subjects in the YS group included volunteers aged from 22 to 40 years old, and in the OS group, volunteers more than 60 years of age were recruited (range 61–72 years). All participants were right-handedly tested with the Edinburgh inventory (Oldfield, 1971). Exclusion criteria included those associated with MRI use (metallic implants, claustrophobia, etc.), the presence of neurological, cognitive, or visual dysfunction, current pharmacological treatment modulating the central nervous system, and abnormal findings on the participants’ structural MRI scan.

Volunteers were mostly recruited from the University of Navarra’s studentship and alumni. All participants had higher education; we requested information about studies or professions using their ability to visualize and spatial judgment, finding engineering training and profession in similar percentage in both groups (percentage of professions in YS was as follows: Life Sciences 43%, Education and Psychology 21%, Social Sciences 24%, and Engineering 12%; percentage of professions in OS was as follows: Life Sciences 23%, Education and Psychology 37%, Law and Social Sciences 28%, and Engineering 12%). All but two YS volunteers were research-naive and had never participated in any research study before. The experimental protocol was approved by the University of Navarra Research Ethics Committee. Subjects signed a written informed consent before participating in the study.

### Driving simulator setup and evaluation

The driving simulator setup used in this study (Signos, Prometeo Innovations C) consisted of a PC, a 40-inch TV, the Logitech G25 Driving Wheel, Pedals and Stick, and a racing seat (Supplementary Image 1). The screen was positioned 1 m in front of the driver while seated and displayed a simulated first-person view from the inside of a Toyota Yaris driver’s seat. The driving session consisted of a 40-min evaluation where participants had to follow a set of pre-defined verbal instructions given by an automated voice, like a GPS system (e.g., “at the roundabout and take the second exit”) through a three-stage circuit (Supplementary Image 2) while driving as they would in a real car. The first stage took place in an urban environment that included traffic lights, different speed limits, pedestrians crossing the street, slow traffic, and a roundabout crossing that lasted for approximately 2.4 km. The second stage was done on a highway where participants had to drive for 11.6 km (round trip) with a 120 km/h speed limit. In the third stage, participants had to drive through a two-lane mountain road (9.6 km, roundtrip) with traffic and different speed limits. Prior to the examination, subjects were allowed to practice driving on “free mode” for up to 20 min, in order to get familiarized with the simulator controls and sensitivity to steering, accelerating, and braking. No systematic monitoring was carried out for simulator sickness symptoms during the experiment. The participants were requested to stop the trial in case they experienced any uncomfortable symptoms. They were asked to communicate any symptoms or simulator sickness and their intensity after the experiment.

A total of 27 telemetric parameters were registered during each driving evaluation, which were related to how fast the participant was driving (total session time, % of time moving, % of time above the speed limit, and mean speed at 40, 50, 80, 100, and 120 km/h limit areas), pedal management (time with gas pedal pressed over 75%, % of time with gas pedal pressed over 75%, time braking, % of time braking, time with brake pedal pressed over 75%, % of time with brake pedal pressed over 75%), number of >5 s brakes (brake pedal pressed over 5 s), number of >10 s brakes (brake pedal pressed over 10 s), steering (number of 60°–90°, 90°–180°, 180°–270°, and 270°–360° steers in 0.5 s), and traffic violations (two wheel sidewalk invasion, collision with other cars or objects, yellow or red traffic light skips, runovers, and restarts). After each driving session, a log file including these parameters was created, from which data were then extracted and analyzed.

### Functional magnetic resonance imaging experimental setup and design

**FIGURE 1:**
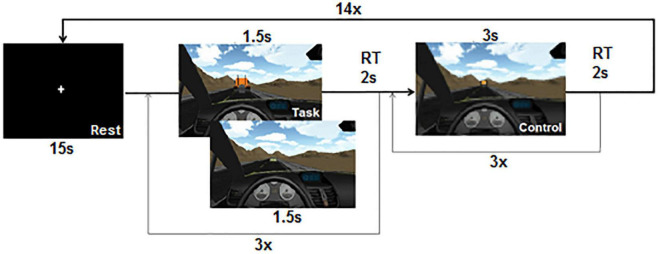
Egocentric distance perception functional magnetic resonance imaging (fMRI) paradigm. At each TASK trial participants were instructed to answer in which of the images the vehicle was the furthest (first or second). RT, response time window.

The EDP task was built using the Unity (v5, 2015) game engine. The objective of this experiment was to determine the participants’ ability to estimate the distance of a vehicle from the observer and to determine whether it was closer or farther when compared to another vehicle within a naturalistic driving scenario (Figure 1). Each cycle (14 in total) began a rest period (Rest) where subjects were asked to keep their gaze fixed on a central cross for 15 s. They were then presented with a block of the Task condition, which consisted of a series of three trials each of which contained two images. Each image represented the point of view of a driver inside a Peugeot 207, driving on a straight road with a mountainous background and was presented for 1,500 ms. In the first image (reference image), a vehicle (car or truck, to avoid participants using stimuli size as a cue) was presented in front of the observer, on the road at one of the 14 different possible distances (from 10 to 140 virtual meters, in virtual meters, in 10-m intervals) which was assigned pseudo-randomly. Then, a second image was presented but with a new vehicle (car or truck) at a shorter or longer distance than the previous vehicle, but never at more than two intervals (10 or 20 m apart) away. At the end of the presentation, a response window of 2,000 ms was left in which the participant had to answer the question “which of the two vehicles was the furthest?” by pressing the first (left-most button) of the button box if he/she thought the first vehicle was farther away, or by pressing the second button (from left to right) if he/she thought the second vehicle was the furthest. Subsequently, a block of the Control condition was presented, where the subject was requested to detect a single image presented during 3,000 ms, which resembled the one in Task, but “pixelated” (by reducing the original resolution from 1,280 × 720p to 128 × 72p) in such a way that it was still possible to recognize the context of the image (driving on a road in a scenario with mountains) but not how far were vehicles from each other. The spatial distribution, chromatic scale, and shapes of objects in the scene were similar but with a lower resolution, which would prevent the participants to estimate the distance between the vehicles. At the end of this image, a 2,000 ms response window was presented where the subject was asked to press any button in response to detecting the stimulus. The duration of each Task and Control block was 15 s, for a total of 45 s per cycle (Rest = 15 s, Task = 15 s, and Control = 15 s) which was repeated 14 times. The total duration of the experiment was 10 min and 30 s. Participants practiced briefly before entering the scanner to be familiarized with the task.

Each of the 42 EDP trials was classified into groups of 14 according to the maximum distance from the observer in each of the comparisons: Close if it was 60 m, Mid if it was 100 m, and Far if it was 140 m away.

### Egocentric distance perception performance variables

In this study, two behavioral variables were individually assessed: Accuracy (AC), defined as the percentage of correct responses, and response time (RT), defined as the average time between the stimulus offset and each of the participants’ correct responses in each trial. Normality and homogeneity tests were performed on both variables.

### Behavioral statistical analysis

To compare values of AC and RT between groups, two mixed ANOVAs (factors Group, with levels YS and OS, and Distance with levels Near [up to 60 m], Mid [up to 100 m], and Far [up to 140 m]) were conducted to test the effect of AC and RT on groups at different distances.

To understand the relationship between the series of variables delivered by our driving simulator, we performed an exploratory factor analysis test which included the 27 telemetric variables in order to find the main factors that could better explain data variance. Specifically, we ran a parallel analysis, with orthogonal rotation (varimax) and a loading threshold of 0.6. Factors obtained by the EFA were then correlated to our EDP performance variables to explore a possible association between EDP and driving in our simulator. All behavioral statistical analyses were conducted using the JASP software (JASP Team, 2021, v0.15).

### Functional magnetic resonance imaging data acquisition and analysis

BOLD fMRI studies were performed on a 3.0 Tesla MR scanner (Siemens TRIO, Germany) using a 16-channel head coil. A total of 210 whole-brain functional volumes using a T2*-weighted gradient echo-planar imaging (EPI) sequence (repetition time/echo time [TR/TE] = 3,000/30 ms, field of view [FOV] = 192 × 192 mm^2^, flip angle = 30°, 48 slices, and resolution = 3 × 3 × 3 mm^3^) were acquired during each session in an interleaved fashion. A total of three initial “dummy” volumes were discarded due to scanner stabilization.

The anatomical image was obtained using a whole-brain T1-weighted MPRAGE sequence [TR/TE = 1620/3 ms, inversion time (TI) = 950 ms, FOV = 250 × 187 × 160 mm^3^, flip angle = 15°, 160 slices, and resolution = 1 × 1 × 1 mm^3^]. No fat suppression was employed.

Mass univariate data analysis was done using SPM12 (r6225, Wellcome Department of Imaging Neuroscience, UCL, London). To detect and correct severe motion or artifacts, we completed preliminary visual checks using Check Reg. Conventional preprocessing pipeline steps were followed. First, slice time correction was applied, taking slice 25 as the reference slice. EPI images were then motion-corrected and realigned to the first volume of the series and co-registered to the anatomical image. Functional and anatomical images were then normalized to the coordinates of the Montreal Neurological Institute (MNI) template, version ICBM-152. A three-dimensional Gaussian smoothing kernel of 8 mm full width at half maximum (FWHM) was applied to the EPI images.

Statistical analysis was performed following the General Linear Model (GLM) and modeled with the canonical double-gamma hemodynamic response function (HRF). At the first-level analysis, two Conditions (Task and Control) were modeled for all participants in both Groups (YS and OS). In both Conditions, only the timeframe between the stimulus onset and the subsequent 2 s was modeled into the design matrix. The 24-parameter Volterra expansion motion regressors (Friston et al., 1996) were included in the design matrix to control for head movements generated during image acquisition. All participants’ Task and Control contrasts were obtained and exported for further analysis.

Second-level analyses comparing Task > Control between groups were done using a two-factor ANOVA, where task-related differences in brain activity between Groups (YS > OS, OS > YS) and mutual coactivations (Global conjunction, positive and negative YS∩OS) were obtained using a primary threshold of *p* < 0.001 and corrected for multiple comparisons using the Family-wise error (FWE, *p* < 0.05) method at the cluster level Anatomical cerebral activations were defined at the peak activation maxima.

To evaluate the effect of performance on brain activations, we included AC and RT as second-level covariates.

### Regions of interest definition and functional connectivity analysis

Regions of interest (ROI) were obtained by extracting the coordinates of peak activations in each significant cluster from the fMRI analysis and selecting its corresponding ROI from CONN’s default atlas (Harvard-Oxford cortical and subcortical atlas and the AAL atlas for cerebellar areas).

Non-smoothed images were used and analyzed using the CONN 16.b Functional Connectivity Toolbox (Whitfield-Gabrieli and Nieto-Castanon, 2012). Our pipeline analysis included the following steps: BOLD data smoothing (8 mm), despiking, and bandpass filtering (0.008-inf) in order to avoid “spillage” between blocks and conditions. White matter, cerebrospinal fluid, and movement parameters were treated as nuisance regressors. Also, main Condition effects were removed from the signal in order to avoid task-related coactivation effects. Then, we conducted a gPPI-based ROI-to-ROI analysis that allowed us to measure changes in functional connectivity that covaried with the experimental factors.

## Results

### Egocentric distance perception performance

**FIGURE 2:**
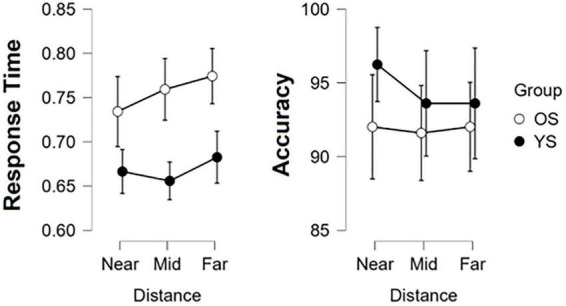
Egocentric distance perception performance results. Response Time **(left)** and Accuracy **(right)** were compared by groups (YS, young subjects and OS, older subjects) and within Distance; circles indicate the mean and whiskers the 95% confidence interval.

Results from our mixed two-way ANOVA analysis for AC and RT are summarized in Figure 2. Despite consistent higher RT across conditions in the OS, no significant effects of Group [*F*(1,34) = 1.254, *p* = 0.271], Distance [*F*(2,68) = 0.555, *p* = 0.576], or interactions [*F*(2,68) = 0.406, *p* = 0.668] were found. In AC, non-significant effects of Group [*F*(1,34) = 3.28, *p* = 0.079] and Distance [*F*(2,68) = 2.059, *p* = 0.135], as well as their interactions [*F*(2,68) = 0.799, *p* = 0.454], were found.

### Driving simulator results

Results of the driving simulator performance are detailed in Table 1. The exploratory factor analysis indicated that 22 variables (with no cross-loadings) surpassed the loading threshold, which was intentionally set high due to the relative number of variables. Bartlett’s Test of Sphericity was significant (χ^2^ = 1313.283, *p* < 0.001), demonstrating the adequacy of our data. Five uncorrelated factors were identified, which explained 72.2% of the variability of our data. The relationship between variables within each factor allowed for appropriate labeling: driving Speed, which included variables related to the time it took the participant to finish the driving session, average speed while driving, and yellow light skips; Steering, which included sudden steering at any angle; Dexterity, which included variables related to traffic violations and speeding; Braking, associated with braking variables; and Hard Braking, which was related with hard brake pressing parameters. From these factors and their variables, we created composite scores to compare results between groups and explore an association with performance in the EDP task. These scores were based on the sum of the mean value of every variable in each factor, weighted by its loading value.

**TABLE 1:** Driving simulator factor and telemetry data.

	YS	OS	YS vs. OS		
	M ± SD	M ± SD	*W*-test	*P*-value	Effect size
Speed	3.051 ± 4.35	−3.41 ± 4.071	42	<0.001*	−0.74
Total session time	2023.763 ± 225.278	2426.559 ± 330.012	279	<0.001*	0.728
% Time above speed limit	4.619 ± 3.561	1.755 ± 1.187	57	<0.001*	−0.647
Mean speed at 40 km/h zone	31.607 ± 3.537	26.844 ± 6.16	76	0.007	−0.529
Mean speed at 50 km/h zone	16.65 ± 3.076	14.376 ± 1.888	84	0.015	−0.48
Mean speed at 80 km/h zone	50.503 ± 5.813	43.317 ± 5.842	60	0.001*	−0.628
Mean speed at 100 km/h zone	49.007 ± 9.479	38.714 ± 5.413	59	<0.001*	−0.635
Mean speed at 120 km/h zone	95.11 ± 12.156	76.155 ± 11.986	44	<0.001*	−0.728
Yellow traffic light skips	1.842 ± 1.344	3.235 ± 1.985	239.5	0.012	0.483
Steering	−0.629 ± 1.078	0.703 ± 4.545	193	0.326	0.195
60–90° steers in 0.5 s	58.368 ± 18.031	71 ± 42.27	194	0.31	0.201
90–180° steers in 0.5 s	38.474 ± 14.331	46.941 ± 37.046	171	0.775	0.059
180–270° steers in 0.5 s	2.842 ± 2.588	4.824 ± 8.338	180.5	0.55	0.118
270–360° steers in 0.5 s	0.105 ± 0.315	0.588 ± 1.326	185.5	0.251	0.149
Dexterity	0.033 ± 3.511	−0.037 ± 1.845	192	0.342	0.189
Time w/gas pedal > 75%	61.963 ± 65.315	48.382 ± 36.503	157.5	0.912	−0.025
% Time w/gas pedal > 75%	1.684 ± 1.94	0.989 ± 0.769	142	0.547	−0.121
Collisions w/other cars	0.526 ± 0.905	0.412 ± 0.712	155.5	0.83	−0.037
Two wheel sidewalk invasion	58 ± 25.153	71.588 ± 17.836	241	0.012	0.492
Collisions w/other objects	3.895 ± 3.799	5 ± 3.791	198.5	0.244	0.229
Braking	0.18 ± 1.354	−0.202 ± 2.109	124	0.241	−0.232
Time braking	261.132 ± 82.111	260.412 ± 154.588	139	0.486	−0.139
% Time braking	13.061 ± 4.473	10.733 ± 5.735	104.5	0.073	−0.353
Hard braking	−0.07 ± 1.4	0.078 ± 3.176	129	0.311	−0.201
% Time moving	87.648 ± 2.953	88.038 ± 5.655	200.5	0.222	0.241
Time w/brake pedal > 75%	3.632 ± 7.654	5.324 ± 16.622	171.5	0.736	0.062
% Time w/brake pedal > 75%	0.091 ± 0.189	0.113 ± 0.357	163	0.971	0.009
>5 s brakes	2.421 ± 1.953	1.824 ± 1.286	140	0.495	−0.133
>10 s brakes	6.684 ± 2.907	4.588 ± 3.001	96	0.038	−0.406
Red traffic light skips	1.895 ± 2.158	3.824 ± 6.55	207	0.139	0.282
Run overs	0.158 ± 0.501	0.235 ± 0.437	180.5	0.365	0.118
Restarts	5.158 ± 0.834	4.412 ± 0.618	82.5	0.008	−0.489

Mann–Whitney *U* test. Effect size by rank-biserial correlation. *Significant values after Bonferroni correction. YS, young subjects; OS, older subjects.

When compared by groups, only the factor Speed showed a significant difference between YS and OS. Within this factor, the YS showed a significantly lower total session time, higher % of time above the speed limit, and higher mean speed at 80 km/h, 100 km/h, and 120 km/h zones. These results were corrected for multiple comparisons using the Bonferroni method.

### Relationship between egocentric distance perception performance and driving factors

Pearson correlations between EDP performance variables (RT and AC) and driving factors (Speed, Steering, Dexterity, Braking, and Hard Braking) showed a significant association between AC and Hard Braking (*r* = −0.673, *p* < 0.001), where higher AC correlated with lower Hard Braking scores (less time and % of time pressing with brake pedal beyond 75%).

### Functional magnetic resonance imaging analysis results

**FIGURE 3:**
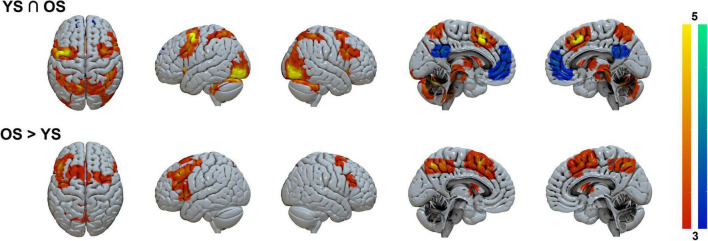
Task > Control shared and differential activation patterns between groups. Color-coded activation (hot) and deactivation (winter) parametrical maps. Clusters were FWE-corrected at *p* < 0.05. YS, young subjects; OS, older subjects.

Conjunction analysis for activations (Task > Control contrast) between groups (Figure 3 and Table 2) showed bilateral precentral, supplementary motor area (SMA), and posterior parietal coactivations, along with activity in occipital, cerebellar, basal ganglia, thalamic, and brainstem areas. Deactivations (Control > Task contrast) were also found in default mode network (DMN) regions (posterior cingulate and medial frontal cortex).

**TABLE 2:** Shared and differential activation in Task > Control contrast.

			MNI coordinates			
Contrast	Cluster size	Region	*x*	*y*	*z*	*t*-Value
YS ∩ OS (pos)	9075	Right middle occipital gyrus	32	−70	28	8.23
		Right supramarginal gyrus	44	−36	44	7.82
		Right inferior occipital gyrus	38	−80	−6	7.66
		Right inferior parietal lobule	34	−44	42	7.1
		Right cerebellum (Lobule VI)	28	−60	−28	7.02
		Right superior parietal lobule	20	−62	52	6.61
		Right cerebellum (Crus 1)	44	−54	−32	5.67
		Left cerebellum (Crus 1)	−6	−72	−26	5.09
		Right inferior temporal gyrus	52	−50	−8	4.92
		Right fusiform gyrus	40	−48	−18	4.89
		Right cerebellum (Lobule VI)	6	−70	−24	4.62
	8554	Left inferior occipital gyrus	−36	−84	−10	8.7
		Left inferior parietal lobule	−34	−46	46	8.44
		Left superior occipital gyrus	−26	−68	32	7.13
		Left middle occipital gyrus	−40	−66	2	6.88
		Left precuneus	−14	−62	54	6.74
		Left fusiform gyrus	−38	−58	−14	6.37
		Left cerebellum (Lobule VI)	−34	−50	−30	5.4
		Left cerebellum (Lobule IX)	−12	−46	−48	4.69
		Left superior parietal lobule	−32	−58	64	4.28
	3325	Left SMA	0	18	48	7.22
		Left inferior frontal gyrus (p. Opercularis)	−44	4	26	6.85
		Left precentral gyrus	−42	2	54	5.94
		Left middle frontal gyrus	−36	2	64	5.62
		Left middle cingulate cortex	−12	22	34	4.7
		Left SMA	−8	8	58	4.53
		Left inferior frontal gyrus (p. Triangularis)	−58	20	28	3.91
		Right middle cingulate cortex	10	26	34	3.89
		Left frontal superior gyrus	−22	2	74	3.41
	2575	Left insula	−32	20	2	8.39
		Right insula	32	22	2	7.65
		Left putamen	−20	6	6	5.18
		Left thalamus	−12	−10	4	4.9
		Left thalamus	−18	−8	0	4.85
		Right thalamus	10	−6	4	4.24
		Right pallidum	14	8	−2	4.11
	954	Right inferior frontal gyrus (p. Opercularis)	46	8	26	6.98
	573	Right precentral gyrus	34	2	52	5.2
	429	Brainstem	8	−26	−8	4.86
		Brainstem	−4	−24	−12	4.61
YS ∩ OS (neg)	1099	Left anterior cingulate cortex	−8	50	0	5.19
		Right superior frontal medial gyrus	6	58	10	4.75
		Left superior frontal medial gyrus	−8	58	18	4.69
	371	Right posterior cingulate cortex	8	−48	28	4.58
		Left posterior cingulate cortex	−8	−48	30	4.57
OS > YS	5509	Left precentral gyrus	−38	6	38	6.75
		Left middle frontal gyrus	−42	22	40	5.71
		Left SMA	−6	16	54	5.39
		Left inferior frontal gyrus (p. Triangularis)	−52	16	0	4.87
		Right superior frontal gyrus	14	12	52	4.74
		Left superior frontal medial gyrus	−8	26	40	4.72
		Left superior frontal gyrus	−18	10	48	4.63
		Left precentral gyrus	36	0	52	4.47
		Right precentral gyrus	32	−2	48	4.39
		Right middle cingulate cortex	8	20	38	4.27
		Right SMA	6	−4	54	4.18
	967	Left precuneus	−2	−64	50	5.09
		Right precuneus	10	−68	52	4.14
		Left inferior parietal lobule	−28	−54	40	3.92
		Left superior parietal lobule	−22	−66	44	3.84
	399	Right inferior frontal gyrus (p. Opercularis)	42	18	30	4.76
	383	Right pallidum	14	0	6	4.99
	322	Left putamen	−22	10	8	4.28
		Left pallidum	−12	0	4	4.28

Clusters are corrected for multiple comparisons using FWE. YS, young subjects; OS, older subjects.

Significant differential activations between groups were only found in the contrast OS > YS where the OS hyperactivated bilateral frontal (mainly the SMA and prefrontal cortex) and parietal regions (precuneus), as well as in the basal ganglia (putamen and pallidum).

When exploring the effect of performance (AC and RT), we found no significant correlation with brain activations.

### Connectivity analysis results

**FIGURE 4:**
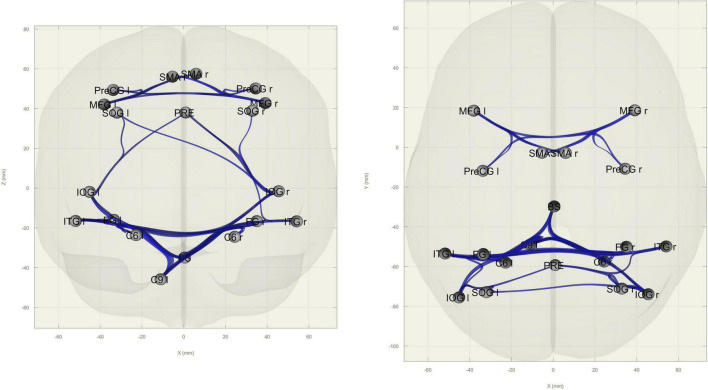
Between-group differences in ROI-to-ROI functional connectivity. Blue edges indicate higher connectivity in older subjects when compared to younger subjects between nodes (in gray). Edges are FDR-corrected at *p* < 0.05 at the cluster level (connection threshold: *p* < 0.05). **Left:** coronal view; **right:** axial view.

ROI-to-ROI functional connectivity analysis revealed increased connectivity in the OS group within nodes in the occipital cortex (middle and superior occipital gyri, fusiform cortex) and between other parietal (precuneus), cerebellar (lobules VI and IX), and brainstem nodes as well as within frontal (SMA, precentral, and middle frontal gyri) nodes (Figure 4).

## Discussion

Results from our driving simulator show a previously observed driving pattern in the elderly, characterized by generalized slower driving. Vehicle telemetry data allowed us to identify five different driving factors or behaviors. The relative perception of distances remains largely intact with advancing age, with similar performance in both groups of age. To associate our simulator and behavioral data, we found that task accuracy was negatively correlated to the Hard Braking behavior (better AC, less hard brake pedal pressing). Functionally, EDP was related in both groups to activation of frontoparietal, cerebellar, and subcortical structures and deactivation of the DMN. Meanwhile, the OS hyperactivated prefrontal, precentral, and posterior parietal regions, along with the basal ganglia. Our connectivity analysis presented increased connectivity within the frontal and a parieto-occipital-cerebellar network exclusively in the elderly.

### Egocentric distance perception is preserved in older adults

When comparing the average values of precision in the EDP, we found no differences between age groups; these results held even across the three different egocentric distances. These findings are in accordance with previous research on EDP and aging, which indicate that this function is preserved in accordance with some studies where this effect remains even when accounting for attentional differences between age groups (Bian and Andersen, 2013) or could even improve by having more experience as people grow older (Gajewski et al., 2015; Wallin et al., 2017).

It is important to note that when participants estimate distances, they might also do it from the allocentric frame of reference where the observer’s position is irrelevant to distance estimation (e.g., “the truck is behind the car”). Allocentric distance perception has not been as thoroughly studied as its egocentric counterpart, especially in older adults; however, it has been found that allocentric navigation and wayfinding might be impaired in healthy aging, particularly in 3D scenes (Colombo et al., 2017; Ladyka-Wojcik and Barense, 2021).

### Driving behavior and egocentric distance perception

The main significant result from telemetric data was that older adults drove at a lower speed when compared to younger adults, a usual finding when evaluating driving behaviors in aging (Shinar et al., 2005; Cantin et al., 2009; Thompson et al., 2012; Keay et al., 2013; Eudave et al., 2018; Wechsler et al., 2018). In an attempt to associate EDP and driving performance, we found that higher accuracy in the EDP task is associated with a reduction in the Hard Braking behavior in our simulator. Although this relationship has not been reported before, it is possible that preserved EDP functioning is necessary to correctly estimate how distant other moving objects, such as cars, are from the viewer, which would then help elicit the correct response to a sudden decrease in that distance, such as fully pressing the brake pedal.

### Egocentric distance perception functional correlates

Common activation between young and older adults when performing the egocentric distance perception task revealed the use of both the ventral (middle and inferior occipital gyri, and fusiform gyrus) and dorsal (inferior parietal lobe and posterior parietal cortex) visual pathways. The activation of these areas may correspond to merely visual aspects, such as the distance that separates two objects (Berryhill, 2009), if the object is closer or further away (Amit et al., 2012) or if the stimulus to be compared is an object or a scene (Persichetti and Dilks, 2016). Activations of bilateral frontal cortical regions are also found to be active during short-term memory encoding and retrieval of 3D objects (Baumann et al., 2010), useful when reproducing perceived distances (Wiener et al., 2016). DMN involvement was observed in this EDP task by deactivating the precuneus and medial frontal cortex, regions that can participate in the projection of oneself in time and space, thus helping in distance estimation (Buckner and Carroll, 2007; Peer et al., 2015). A definitive role of the cerebellum in EDP is unknown, although it has been related to other aspects of visuospatial cognition. The cerebellum takes part in the representation of the body in space by participating in hippocampal spatial navigation. Using the L7-PKCI transgenic model mice, whose protein kinase C activity is specifically inhibited in the Purkinje cells, a deficit in the use of self-motion cues and inability to detect the relevant features of the environment were found without motor coordination deficits (Rochefort et al., 2013; Lefort et al., 2015). Lastly, our EDP task also involved the basal ganglia. Classical studies have found that the basal ganglia (specifically, caudate nucleus) are involved in the egocentric frame of reference (Cook and Kesner, 1988). The role of the putamen in the computation of egocentric coordinates was initially demonstrated in neurophysiological studies (Cavada and Goldman-Rakic, 1991; Graziano and Gross, 1993) and may modulate the degree to which participants overestimate or underestimate distances (Wiener et al., 2016). The reciprocal relationship of the caudate nucleus and putamen with the cerebral cortex mediates egocentric memory, the relationship between the individual and its environment (White, 1997).

### Frontoparietal activation as attempted compensation in older adults

The hyperactivation pattern in older adults showed greater recruitment of parietal and frontal areas, as well as the basal ganglia. This effect corresponds to findings found in other cognitive paradigms where older adults use more frontoparietal resources (Grady et al., 2016), including visuospatial paradigms (Madden et al., 2017). This increase in cortical activity tends to be proportional with age (Kennedy et al., 2015), and despite it being predominantly prefrontal and right-sided, this pattern can also be more diffuse and multimodal, employing different associative areas. Typically, these changes in brain activity have been correlated to task performance, accomplishing a “successful” compensation (Cabeza and Dennis, 2013). However, in our study, this hyperactivation in older adults did not correlate with parameters of task performance, a finding that could be attributed to “attempted” compensation, where activity is instead related to structural brain decline and task demands. In this study, the EDP task could have been more demanding in older adults given limited neural resources or cognitive processing capacity (Scheller et al., 2014).

Areas belonging to this hyperactivation pattern in the OS correspond to findings in functional connectivity, where we detected an increase between frontal nodes and between parietal, visual, and cerebellar nodes when compared to younger adults. To the best of our knowledge, there are no studies examining the functional connectivity of the EDP in both young and older adults. In monkeys, it has been shown that the posterior parietal cortex projects to the cerebellar hemispheres *via* the pontine nucleus, which could contribute substantially to multisensory integration (Glickstein, 2003). It is possible that this increase in connectivity in the OA is part of a compensatory network, necessary to sustain cognitive skills. In a visual working memory experiment, Burianová et al. (2015) found that while young adults are able to modulate baseline connectivity as cognitive load increases, older adults are not, and instead display a predominantly frontal compensatory network which also includes the inferior parietal lobe. This could be attributed to an increase in connectivity within (frontoparietal) networks that comes with aging, a parameter that has been found to predict task skills (Grady et al., 2016). Additionally, this increase in connectivity might also be a consequence of the depletion of the participants’ cognitive reserve, which also increases with task difficulty (Bastin et al., 2012). This supports our “functional compensation” findings, since the EDP task was relatively easy allowing these extra resources to intervene and possibly sustain young-like performance.

### Limitations and future direction

As with other driving simulator studies, our simulator might have introduced bias, since it does not exactly portray the act and experience of real driving. Also, our driving task might have proven difficult for some drivers since it required navigation in a complex environment while also following instructions, with a relatively short training session. Additionally, despite these throwbacks, we believe that this naturalistic approach to driving allowed for reliable telemetry data collection. Also, some of our participants (mostly older adults) experienced mild symptoms of simulator sickness (eye strain and dizziness).

An important caveat is that our task intended to evaluate EDP from a set of static images; however, distance perception is usually carried out in a dynamic fashion, especially in the context of movement, such as car driving, where the subjects’ point of view is constantly changing and updating. Also, future studies should include the evaluation of the allocentric frame of reference which, as stated previously, might be impaired in older adults and be involved in distance perception.

Another limitation and future work of this study is the validation of the factor analysis and its relationship with EDP performance in an independent sample that could not be performed due to the limited sample size in this study. Also, the few female participants did not allow for an analysis of the differences between the sexes.

## Conclusion

In conclusion, performance in egocentric distance perception was similar between young and older adults. Both groups showed consistent accuracies and response times independent of how far the vehicle was from the viewer. Driving in a simulator confirmed previous findings of generally slower driving in older adults. Additionally, five driving behaviors were identified by means of an EFA; from these, Hard Breaking behaviors when driving were associated with how accurate participants were in the EDP task. Neuroimaging results identified the hyperactivation of frontoparietal and basal ganglia in older adults, along with increased connectivity within frontal nodes and between a posterior network comprised of posterior parietal, occipital, and cerebellar nodes.

## Data availability statement

The raw data supporting the conclusions of this article will be made available by the authors, without undue reservation.

## Ethics statement

The studies involving human participants were reviewed and approved by the University of Navarra Research Ethics Committee. The patients/participants provided their written informed consent to participate in this study.

## Author contributions

LE and MP contributed to the conception and design of the study. LE, MM, EL, and MP collected participant data. LE organized data and performed all analyses. LE and MP interpreted the results and wrote the first draft of the manuscript. All authors contributed to the manuscript revision, read, and approved the submitted version.
