# Supplementary figures and images for: Egocentric distance perception in older adults: Results from a functional magnetic resonance imaging and driving simulator study
Source: Front Aging Neurosci. 2022 Oct 6;14:936661. doi: 10.3389/fnagi.2022.936661 (PMC9584650; doi:10.3389/fnagi.2022.936661)

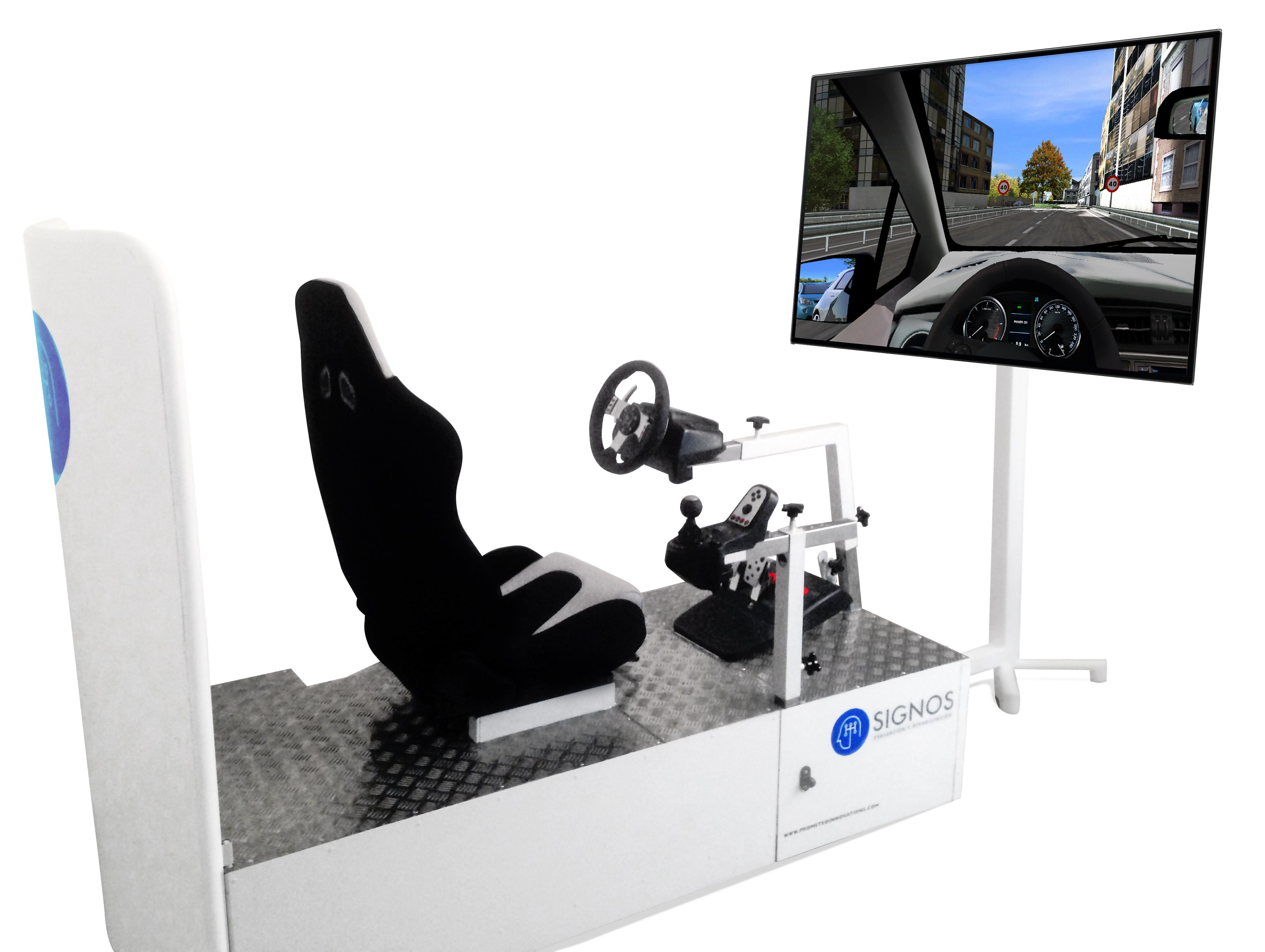

Supplement: Supplementary file 1 [file Image_1.TIFF]

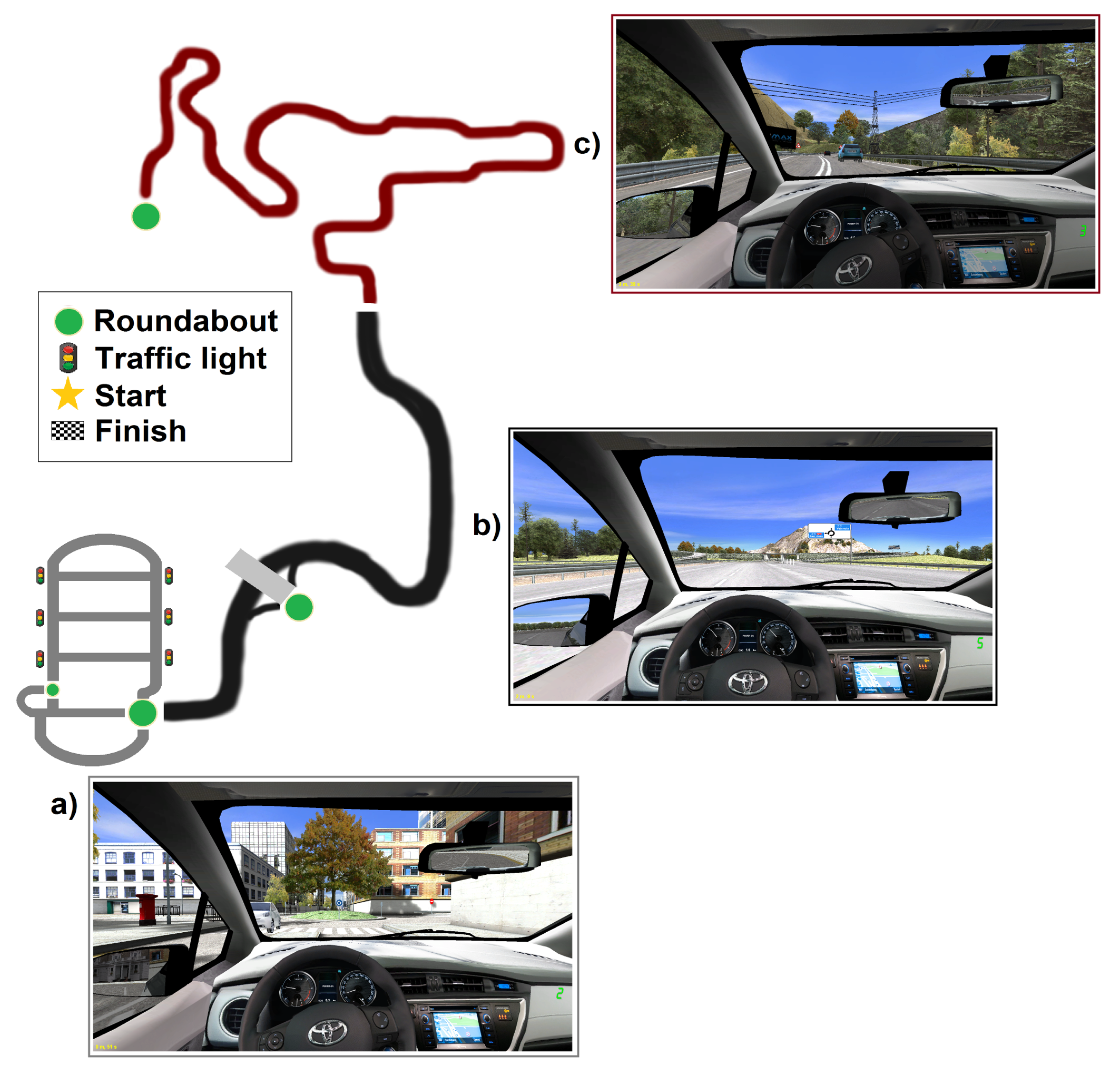

Supplement: Supplementary file 2 [file Image_2.TIF]
